# Altered dynamic functional architecture in type 2 diabetes mellitus

**DOI:** 10.3389/fendo.2022.1117735

**Published:** 2023-01-24

**Authors:** Yifan Li, Mingrui Li, Kui Zhao, Yan Wang, Xin Tan, Chunhong Qin, Yawen Rao, Zhizhong Sun, Limin Ge, Zidong Cao, Yi Liang, Shijun Qiu

**Affiliations:** ^1^ First Clinical Medical College, Guangzhou University of Chinese Medicine, Guangzhou, China; ^2^ Department of Radiology, The First Affiliated Hospital of Guangzhou University of Chinese Medicine, Guangzhou, China; ^3^ Department of Magnetic Resonance Imaging, Zhanjiang First Hospital of Traditional Chinese Medicine, Zhanjiang, China

**Keywords:** type 2 diabetes mellitus, cognitive decline, resting state, functional architecture, dynamic functional stability, dynamic effective connectivity

## Abstract

**Introduction:**

Type 2 diabetes mellitus (T2DM) can accelerate cognitive decline and even dementia so that the underlying mechanism deserves further exploration. In the resting state, brain function is still changing dynamically. At present, it is still unknown whether the dynamic functional connectivity (dFC) between various brain regions is in a stable state. It is necessary to interpret brain changes from a new perspective, that is, the stability of brain architecture.

**Methods:**

In this study, we used a fixed dynamic time scale to explore the stability of dynamic functional architecture in T2DM, then the dynamic effective connectivity (dEC) was used to further explain how information flows through dynamically fluctuating brain architecture in T2DM.

**Result:**

Two brain regions with decreased stability were found including the right supra-marginal gyrus (SMG) and the right median cingulate gyrus (MCG) in T2DM subjects. The dEC variation has increased between the left inferior frontal gyrus (IFG) and the right MCG. The direction of causal flow is from the right MCG to the left IFG.

**Conclusion:**

The combination of stability and dEC can not only show the stability of dynamic functional architecture in brain but also reflect the fluidity of brain information, which is an innovative and interesting attempt in the field of neuroimaging. The changes of dynamic architecture in T2DM patients may present an innovative perspective and explanation for their cognitive decline.

## 1 Introduction

The incidence of type 2 diabetes mellitus (T2DM) is sharply increasing worldwide (1, 2). T2DM has crucial effects on cognition (3), and evidence has shown that T2DM is a generally acknowledged risk factor for Alzheimer’s disease (AD) (4, 5). Some researchers have classified AD as type 3 diabetes (6), as there are some shared pathological mechanisms between T2DM and AD, including insulin resistance, mitochondrial dysfunction, and advanced glycation end products. Studies have shown that T2DM subjects have cognitive decline in the subclinical stage, such as executive function and memory and attention decline (7, 8). The early stage of cognitive decline in T2DM is a gradual and imperceptible process, and it is not always noticeable. Early assessment of cognitive function in T2DM patients is of great clinical significance for early prevention and intervention.

Recently, neuroimaging biomarkers have commonly been used for the early detection of numerous neurological and psychiatric diseases (9, 10). Resting-state functional magnetic resonance imaging (rs-fMRI) can sensitively capture the subtle functional alteration in the brain by collecting the blood oxygenation level-dependent (BOLD) signal in a non-invasive way (11). At this point, existing studies have shown that T2DM patients have abnormal regional and global functional changes, which is closely relevant to the decline of cognitive function in multiple aspects. A recent study revealed that reduced regional gray matter volume and increased functional connectivity (FC) may reveal the neurobiological mechanism underlying cognitive impairment in early-onset T2DM patients (12). Another study showed that abnormal volumes of hippocampal subregions in T2DM patients were associated with memory function, suggesting that reduced volumes of specific hippocampal subregions may be an underlying mechanism of memory dysfunction (13). At the same time, it has previously been shown that brain networks are time-variant during MRI scan (14). The brain is a temporally dynamic system. Even during a short scanning session, cognitive states and FC are continuously evolving (15). The most common approach is dynamic functional connectivity (dFC), which focuses on the distribution patterns and dynamics of signal fluctuations in neuronal activity (16–19). These reported developments in the field of dynamic network neuroscience have enabled quantifying time-variant changes in neural network connectivity, state transition, and functional organization and underscored the needs of revealing brain dynamic features. However, it is not known whether the dynamic pattern is in a steady state, which may be related to changes in cognition (20).

An important feature of consciousness is stability, characterized by the consistency of distributed neural activity and connectivity patterns over time (21). Functional stability can assist in the maintenance of brain function normally in dynamic changes and manifest from a dynamic viewpoint how functional organizations adapt to accomplish complicated natural tasks (22). Yan first clarified the stability of dFC in the human brain, that is, the stability distribution of functional architecture (20). Yan found that functional stability is unevenly distributed in the cerebral cortex and regions with strong stability were mainly distributed in the default mode network. Another study showed that amyotrophic lateral sclerosis patients had abnormal stability of brain dynamic functional architecture, which was relevant to the severity of the disease (23). We speculated that stability of dynamic functional architecture, which captured more sensitive and time-varying information, could be an objective and meaningful method to reveal underlying brain changes and evaluate cognitive function from a new perspective. It is innovative and interesting to investigate stability from a dynamic perspective to reveal the mechanisms of the dynamic functional architecture changes in T2DM.

During cognitive processing, specific messages are transmitted throughout the brain (21). Several complicated cognitive functions depend on constantly coordinating multiple aspects of information in the brain (20). There are continuous endogenous fluctuations in human brain regions that represent underlying brain functional architecture (24). It is essential that the process of transmission is fluid and directional. The related issues are that how information travels and flows through dynamically fluctuating brain functional architecture and whether information transmission is affected by changes in dynamic stability. The dFC represents the temporal consistency between endogenous dynamic fluctuations, but it cannot be used to show direct interactions among brain regions (24). Dynamic effective connectivity (dEC) reflects well the flow of information, emphasizing the directivity of information dissemination in functional dynamic networks. Granger causality analysis (GCA) is commonly used to measure dEC using multiple linear regression, which examines the prediction effect of current time-series values using all the information at some time series in the past (25). In recent years, GCA has been used to elucidate the causal relationship between brain regions (24, 26). However, there is no study combining dEC and stability to measure the dynamic functional architecture and directional connectivity changes in T2DM. In this study, we explored the stability of dynamic functional architecture at a fixed dynamic time scale to elucidate how stability was modified in T2DM. Then, dEC was used to effectively explore information flow in intricate dynamic brain networks, which was an interesting and innovative experiment. Finally, the relationships among the imaging indicators, clinical data, and neurocognitive measurements were further explored. This study was designed to investigate the underlying neuroimaging mechanism of cognitive decline in T2DM patients.

## 2 Materials and methods

### 2.1 Subjects

Fifty T2DM subjects and 58 healthy controls (HCs) were recruited. Details of the subjects are shown in **Table 1**. All subjects met the following requirements: 1) years of education >6; 2) Han Chinese; 3) right-handed. Subjects meeting the following criteria were excluded: 1) brain organic disease; 2) psychiatric or neurological disease; 3) claustrophobia. The exclusion criteria were consistent with our previous study (8). The T2DM criteria were set based on the American Diabetes Association, which contain the following: 1) A1C greater than or equal to 6.5%; 2) fasting plasma sugar (FPG) greater than or equal to 126 mg/dl; 3) after oral glucose tolerance test, blood sugar level greater than or equal to 200 mg/dl; 4) blood glucose level greater than or equal to 200 mg/dl. Each way usually needs to be repeated on a second day to diagnose T2DM. These examinations were performed by endocrinologists. All T2DM subjects were on insulin therapy *via* insulin pump or insulin injections. Age, sex, and education levels of HCs were matched with T2DM subjects.

**Table 1:** Demographic data and clinical biochemical indicators of all subjects.

	T2DM (n = 50)	HC (n = 58)	Statistics	P value
Age (years, x ± s)	50.60 ± 9.33	48.24 ± 7.43	t = 1.461	0.147
Sex (M/F)	29 (58)	30 (51.7)	χ^2^ = 0.427	0.514
Education (years, x ± s)	10.5 (9,12)	10 (8.5, 12)	z = -0.006	0.995
HbA1c (%)	9.12 ± 2.09	N/A	N/A	N/A
FINS (µIU/mL)	7.12 (4.6875, 10.2675)	N/A	N/A	N/A
FPG (mmol/L)	7.825 (6.58, 8.915)	N/A	N/A	N/A
HOMO-IR	2.3569 (1.5149, 3.7948)	N/A	N/A	N/A
TG (mmol/L)	1.63 (0.975, 2.33)	N/A	N/A	N/A
TC (mmol/L)	4.57 ± 0.99	N/A	N/A	N/A
LDL (mmol/L)	3.045 (2.5875, 3.5525)	N/A	N/A	N/A

HC, healthy control; T2DM, type two diabetes mellitus; HbA1c, hemoglobin A1c; FINS, fasting insulin; FPG, fasting plasma glucose; HOMA-IR, homeostatic model assessment of insulin resistance; TG, triglyceride; TC, total cholesterol; LDL, low-density lipoprotein; N/A, not applicable.

### 2.2 Clinical and cognitive assessments

All subjects’ clinical information containing age, sex, and education level was recorded. Biochemical information included FPG, hemoglobin A1c (HbA1c), fasting insulin (FINS), homeostatic model assessment of insulin resistance (HOMA-IR), total cholesterol (TC), triglyceride (TG), and low-density lipoprotein (LDL) cholesterol levels. Comprehensive cognitive assessments were conducted, which include Montreal Cognitive Assessment (MoCA), Auditory Verbal Learning Test (AVLT, including immediate recall, short-term delayed recall, long-term delayed recall, and recognition), Trail Making Test (TMT, including parts A and B), Grooved Pegboard Test (GPT), Symbol Digit Test (SDT), the Clock Drawing Test (CDT), and the Digit Span Test (DST, including forward and backward).

### 2.3 Data acquisition

Rs-fMRI data were collected on a 3T MRI scanner (GE, SIGNA, USA). Conventional sequences were conducted to screen brain lesions, including T1-weighted and fluid-attenuated inversion recovery images. None of the participants were excluded at this stage. Then, the experimental sequences which contain BOLD and 3D-T1 were conducted for data processing. The scanning parameters of BOLD were as follows: repetition time (TR) = 2,000 ms, echo time (TE) = 30 ms, slices = 36, thickness = 3 mm, field of view (FOV) = 220 mm × 220 mm, acquisition matrix = 64 × 64, and flip angle = 90°. The scanning parameters of 3D-T1 were as follows: TR = 2,000 ms, TE = 2.6 ms, slices = 256, thickness = 1 mm, FOV = 250 mm × 250 mm, acquisition matrix = 256 × 256, flip angle = 12°.

### 2.4 Data preprocessing

Imaging data were processed by using DPABI and SPM on the basis of the MATLAB toolbox (27). The steps of preprocessing were as follows: 1) conversion of the image data formats: all the subjects’ image data in DICOM format were converted to NIFTI format; 2) the functional images of the initial 10 time points were removed to stabilize the signal; 3) slice timing: reduce the difference caused by different image acquisition times; 4) head motion: subjects with head movement more than 2 mm or rotation more than 2° were excluded, and Friston 24 head movement parameters were obtained; 5) covariate of regression: interference covariates including linear trend, Friston 24 head motion parameter, white matter signal, and cerebrospinal fluid signal were regressed from the BOLD signal; 6) functional images were normalized to MNI space using DARTEL; 7) spatial smoothing (6-mm FWHM kernel); 8) band-pass filter (0.01–0.1Hz).

### 2.5 Computation of stability of dynamic functional architecture

The stability of dynamic functional architecture meant the consistency of dFC of a voxel with remaining voxels in the whole brain over time. For a given voxel j, the Pearson’s correlation coefficients between the BOLD signal of voxel j and that of other voxels within the gray matter were calculated. Then, the dFC maps across time windows were generated for voxel j. The stability was quantified with the Kendall’s coefficient of concordance (KCC) of these dFC maps, regarding the time windows as raters.

The specific calculation was as follows:


$$W=\frac{12\text{S}}{K^{2}\left(N^{3}-N\right)}$$

$$S=\sum_{n=1}^{N}R_{n}^{2}-\frac{1}{N}{\left(\sum_{n=1}^{N}R_{n}\right)}^{2}$$

W stands for Kendall’s consistency coefficient, which ranges from 0 to 1. A higher W indicates that the results are more consistent. In other words, the dynamic functional stability of the brain is more stable in the field of neuroimaging. K is the number of sliding windows (raters), and N is the number of dFC between the given voxel and all atlases (respondents). *R_n_* is the sum of the ranks of the nth dFC.

**Figure 1:**
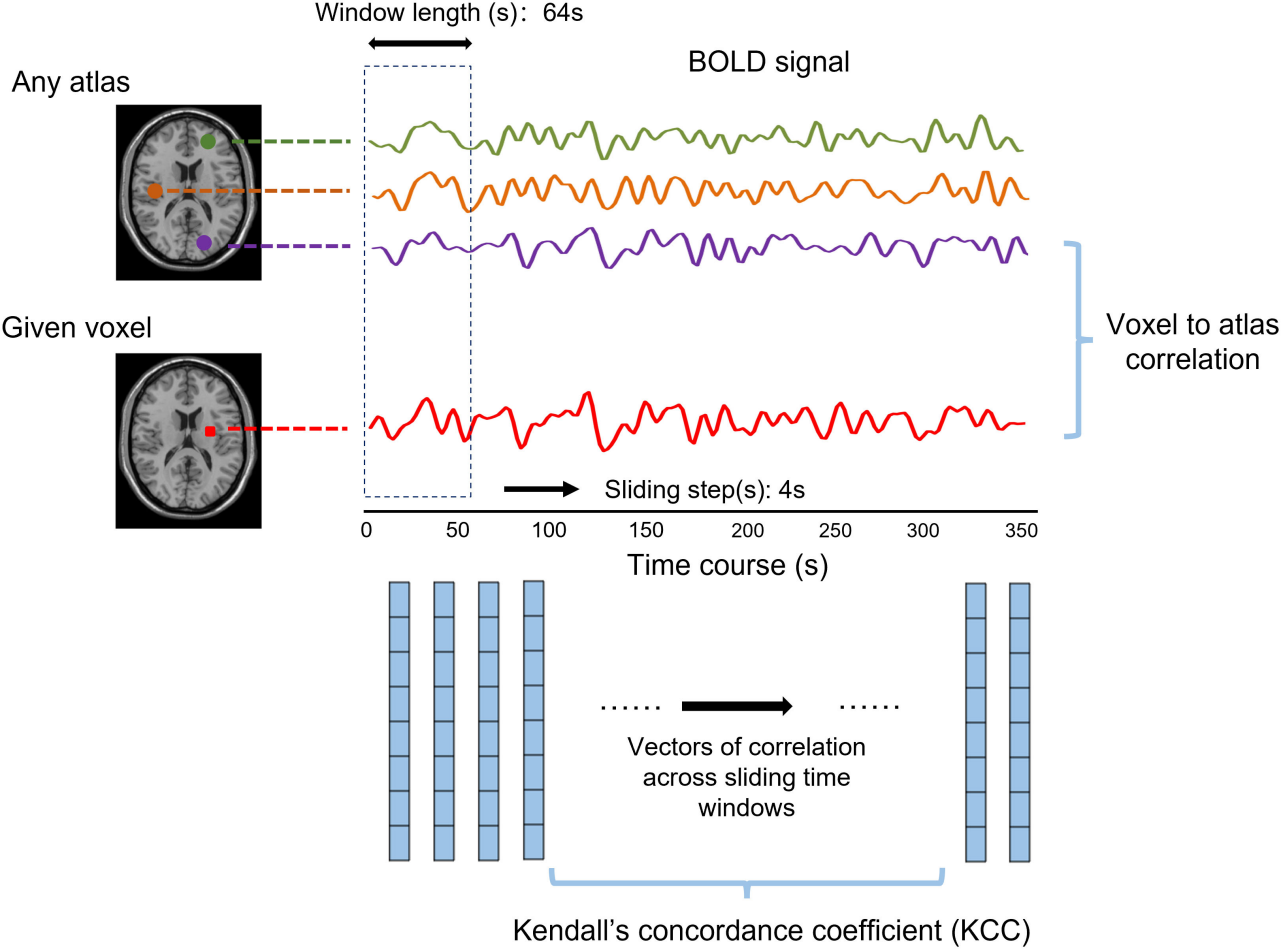
The abridged general view shows the calculation method of dynamic functional stability using the voxel-to-atlas way. The Pearson correlation between a given voxel and any atlas, namely, dynamic functional connectivity (dFC), was calculated for each window. The Kendall’s concordance coefficient was computed based on cross-window dFC to state the functional stability.

In this study, Craddock’s atlas was used to divide the brain into 200 subregions (28). The calculation of the voxel-to-atlas dFC would generate a series of dFC vectors. The KCC of dFC vectors of voxel j was calculated with time windows as raters. The sliding-window approach was used to conduct dFC analysis (14), and the window length was 64 s and the sliding step was 4 s (29) (**Figure 1**). Different window lengths and sliding steps were set to 60 s/2 s and 96 s/8 s, respectively, to verify whether the result was affected by the window lengths and sliding steps.

### 2.6 Computation of dynamic effective connectivity

Brain regions with changed stability in T2DM were identified through the above steps. The regions which were obtained by group comparison of stability were extracted as region of interest (ROI) for further analysis. The dynamic BC toolbox was used to perform the GCA and calculate the dEC (30). The time series of each ROI was defined as x, and the time series of voxels in the whole brain was defined as y. The Granger causality effect between each ROI and each voxel in the whole brain was studied using bivariate coefficient GCA. Similarly, the sliding window method was used to estimate the dynamic GCA. For each subject, the average time course of GCA coefficients for each ROI was extracted and connected into a W × R matrix (where W represents the number of windows and R represents the number of ROIs). The dEC variability of each ROI was calculated by variance over the average time course of the GCA coefficients. The GCA model, based on the time series of the BOLD signal, describes the causal effect of the ROI on the rest of the voxels in the brain.

## 3 Statistical analysis

### 3.1 Demographic and clinical characteristics analysis

Statistical analysis was performed using the Statistical Package for the Social Sciences version 22.0 (SPSS, Chicago, IL, USA). The statistical analysis of demographic and clinical characteristics was consistent with our previous studies (7, 8).

### 3.2 Intergroup comparison of dynamic functional stability and stability-based effective connectivity

The two-sample t-test in DPABI toolbox statistical software was used to compare the stability of the two groups. The gray matter mask automatically generated by preprocessing was used as the registration template, and sex, age, education level, and average head movement parameters were used as covariables to perform Gaussian random field (GRF) correction. Voxel level P< 0.001 and cluster level P< 0.05 were set. Subsequently, the same statistical method was used to conduct a statistical analysis on the dEC maps of the two groups.

### 3.3 The relationship between clinical biochemical indicators and neurocognitive scores

Partial correlation analysis was applied to analyze the correlation between various clinical biochemical indicators and scores of different neurocognitive function scales in the T2DM group after controlling for sex, age, and education level. P< 0.05 was considered statistically significant.

### 3.4 Correlation analysis

The Spearman correlation was conducted between the mean stability/dEC values of significant regions and cognitive function scores in T2DM subjects.

## 4 Results

### 4.1 Demographic, clinical, and cognitive characteristics

T2DM subjects and HCs were well matched across age, sex, and education level. For general cognitive status, the T2DM group performed significantly worse on AVLT (immediate), MoCA, CDT, and GPT(R&L) (P< 0.05) (**Table 2**).

**Table 2:** Neuropsychological result of two groups.

	T2DM (n = 50)	HC (n = 58)	Statistics	*P* value
AVLT (immediate)	20.16 ± 5.10	23.5 (18, 27)	*z* = -2.084	0.037*
AVLT (5 min)	7.56 ± 2.44	8 (7, 10)	*z* = -0.667	0.505
AVLT (20 min)	7.68 ± 2.77	8 (7, 9.5)	*z* = -0.289	0.772
AVLT (recognition)	11 (9, 12)	11 (10, 12)	*z* = -0.470	0.638
TMT-A (s)	53.5 (43.75, 75.25)	53 (41.5, 60)	*z* = -1.159	0.247
TMT-B (s)	45 (33.855, 61)	40 (34.95, 54.05)	*z* = -1.418	0.156
DST (forward)	8 (7,9)	8 (8, 9)	*z* = -1.753	0.080
DST (inverse)	4 (3,5)	4 (3, 4.25)	*z* = -1.328	0.184
CDT score	3 (2,3)	3 (3, 3)	*z* = -2.448	0.014*
MoCA score	26 (23,27.25)	27 (26, 29)	*z* = -3.039	0.002*
GPT (R) (s)	84 (73.125,99.6175)	72 (66.45, 81.85)	*z* = -3.853	<0.000*
GPT (L) (s)	90.35 (78.75,108.5)	80 (75, 89.25)	*z* = -3.381	0.001*

AVLT, Auditory Verbal Learning Test; TMT, Trail Making Test; DST, Digit Span Test; CDT, Clock Drawing Test; MoCA, Montreal Cognitive Assessment; GPT, Grooved Pegboard Test. *P < 0.05.

### 4.2 Intergroup differences in stability in T2DM

**Figure 2:**
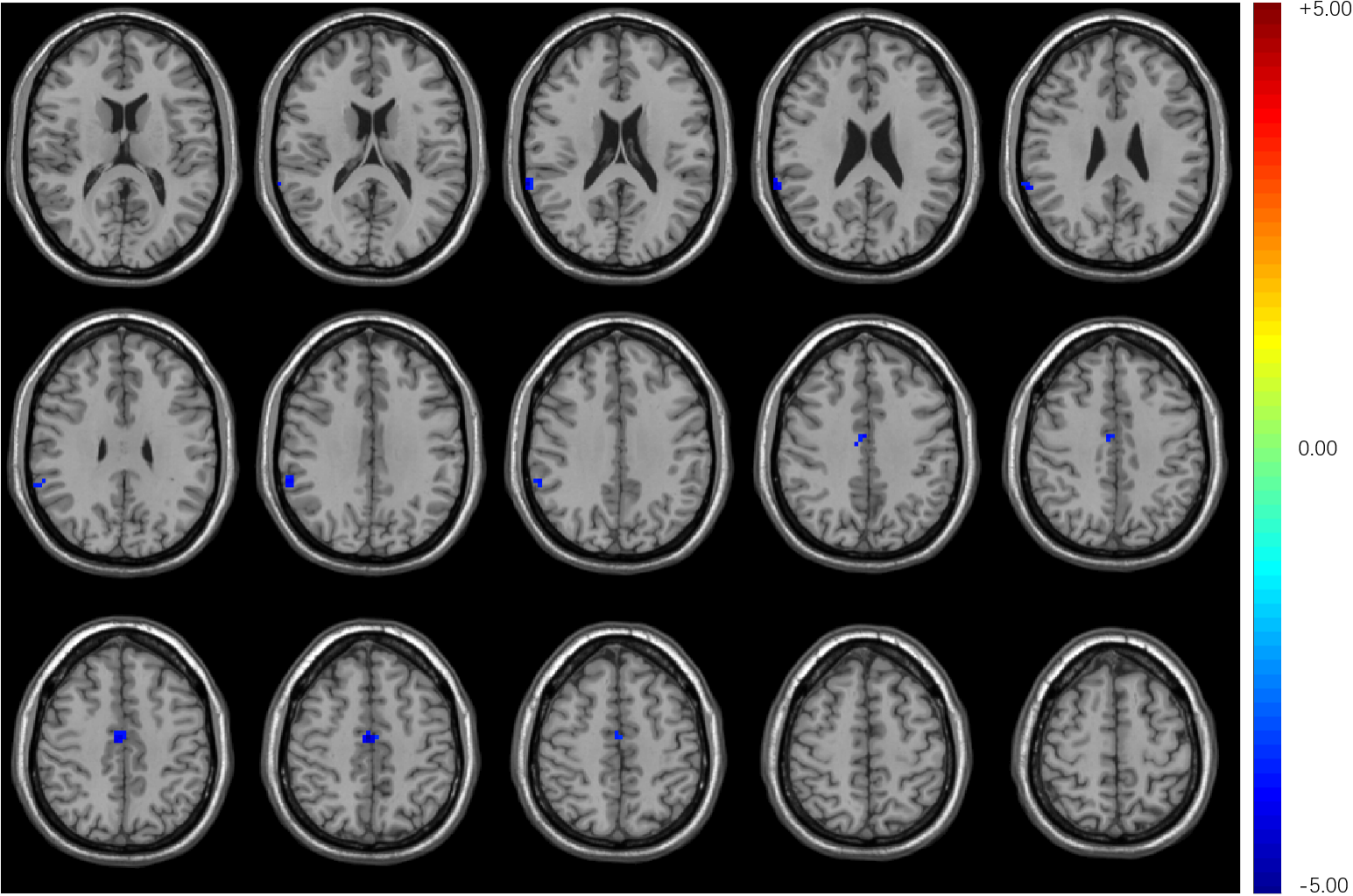
Compared with HCs, T2DM subjects exhibited decreased stability in the right supra-marginal gyrus and right medial cingulate gyrus with a window length of 64 s and a step size of 4 s.

**Figure 3:**
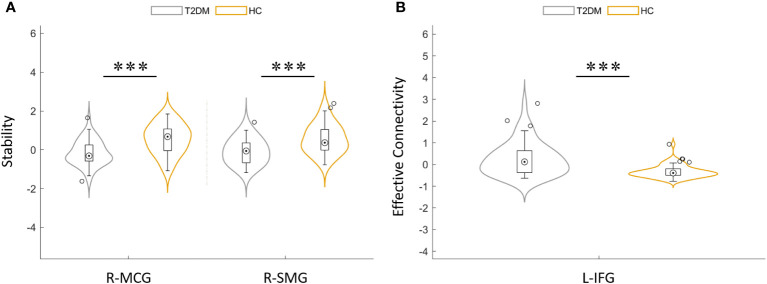
**(A)** Distribution comparison of dynamic functional stability between T2DM group and HCs. **(B)** Distribution comparison of dynamic effective connectivity between the T2DM group and HCs. MCG, median cingulate gyrus; SMG, supra-marginal gyrus; IFG, inferior frontal gyrus; L, left; R, right. ***P< 0.001.

Compared with HCs, T2DM subjects showed decreased stability in the right supra-marginal gyrus (SMG) and the right medial cingulate gyrus (MCG) with window length of 64 s and a step size of 4 s (**Figure 2** and **Table 3**). The distribution comparison of stability was shown in the violin and box plots (**Figure 3A**). To further verify the validity and reliability of the results, validation tests were also processed by using a window length of 60 s and a step size of 2 s and a window length of 96 s and step size of 8 s, respectively (**Supplementary Figures 1**, **2**). The location of the peak MNI point and the cluster were consistent, but the size of the cluster was slightly altered (**Supplementary Table 1**).

**Table 3:** Brain regions with altered stability and EC in T2DM subjects.

Indicator	Cluster	Brain regions	MNI coordinates			Voxels	t-value
			x	y	z		
Stability	1	Right supra-marginal gyrus	69	-42	21	28	-4.5316
	2	Right median cingulum gyrus	3	-12	48	26	-4.746
EC	1	Left inferior frontal gyrus	-48	21	18	23	4.4585

MNI, Montreal Neurological Institute; X, Y, and Z, coordinates of primary peak locations in MNI space.

### 4.3 Intergroup differences in dynamic effective connectivity in T2DM

**Figure 4:**
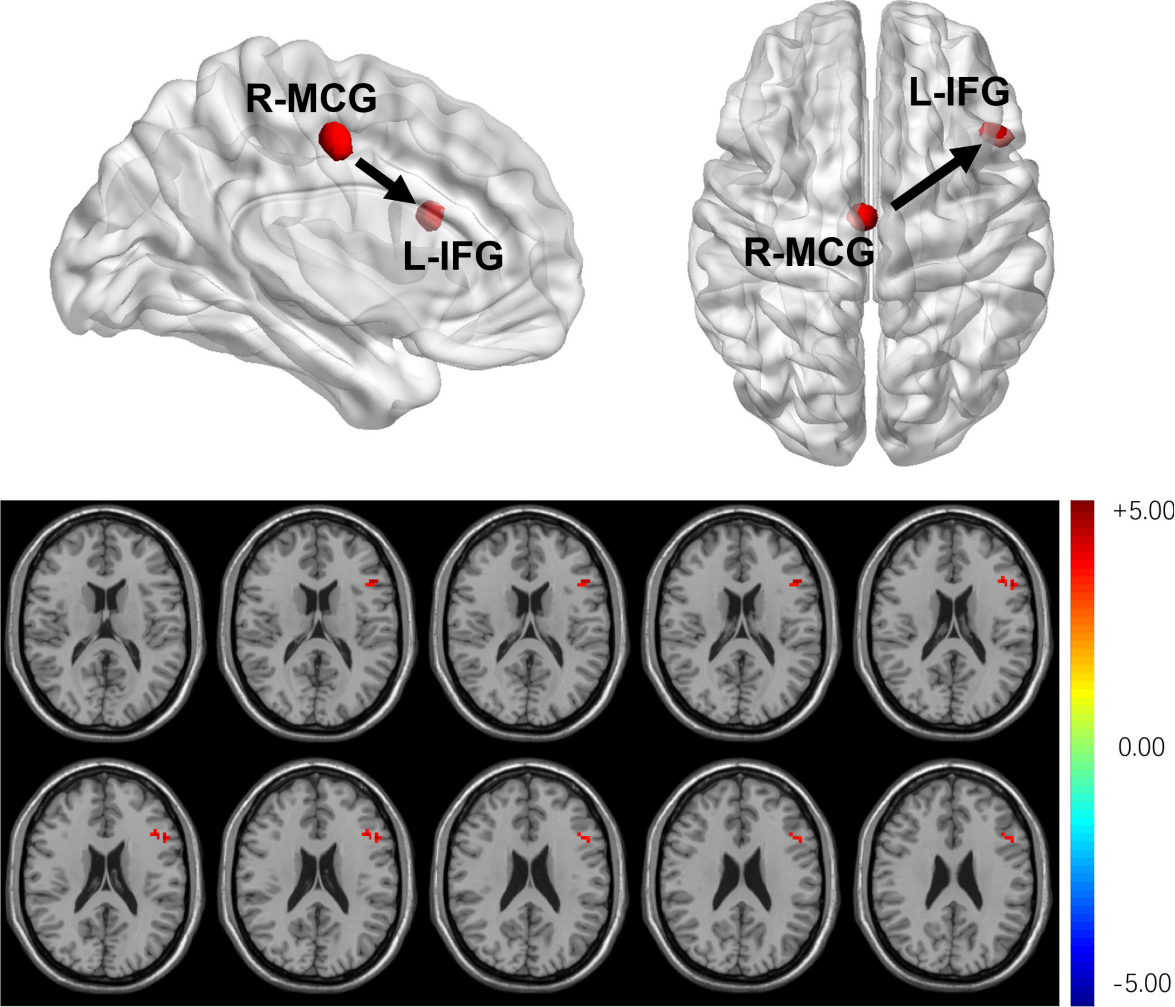
T2DM subjects showed that dEC has increased significantly between the left IFG and the right MCG. The direction of causal flow is from the right MCG to the left IFG.

Compared with HCs, T2DM subjects showed that the dEC variability increased significantly between the left inferior frontal gyrus (IFG) and the right MCG. The direction of causal flow is from the right MCG to the left IFG (**Figure 4** and **Table 3**). The distribution comparison of dEC is shown in the violin and box plots (**Figure 3B**).

### 4.4 Correlation analysis results

Partial correlation analysis found that AVLT immediate memory scores were negatively correlated with HbA1c (r = -0.309, P = 0.035) and LDL (r = -0.399, P = 0.004). However, there were no correlations between altered stability/dEC and scores of the neuropsychological tests.

## 5 Discussion

In the resting state, brain function is still changing dynamically (18). In this study, we explored the dynamic stability and dEC variability of the brain in T2DM. We found that there were two brain regions with decreased stability which contain right the SMG and the right MCG in T2DM subjects. DEC variation increased significantly between the left IFG and the right MCG in the T2DM group, which is further evidence of decreased stability in the brain of T2DM.

### 5.1 Altered functional architecture in T2DM

Stability, which reflects dFC without changing frequently in a continuous state, may provide an effective ability to coordinate information over time and facilitate rapid responses (21). The functional architecture of each region adjusts and changes dynamically and automatically as requested by physical need (31, 32). It is largely unclear whether the functional architecture of the brain in T2DM is stable over time. This study has well proved this point.

In this study, we found that there were two brain regions with decreased stability which contain the right SMG and the right MCG in T2DM subjects. SMG belongs to Brodmann area 40 which is involved in the motor attention and language processes, and lesions in it may lead to receptive aphasia (33). It is also reported that the right SMG was associated with poor melodic perception (34). In addition, the right SMG is a key region controlling spatial attention and working memory (35). The cingulate bundle is the main intermediate bundle, and it is one of the main white matter (WM) structures to transmit information (36). Moreover, the right MCG is contained in the frontoparietal network (FPN), which is involved in top-down attention and control task execution (37). It was found that WM fibers in cingulate gyrus were damaged in AD (38). The amplitude of low-frequency fluctuation (ALFF) is considered to be an effective method for detecting the intensity of spontaneously fluctuating regions and reflecting spontaneous brain activity in the brain. A study suggested that as cognitive function declines, the ALFF value of MCG decreased, which means the weakening in the neural activity of MCG. This finding represents decreased spontaneous neural activity or downregulation of excitability in MCG (36). Regions with high stability receive neural integration across modes and time so that they may conduct more function. Instead, the dynamic stability of these two brain regions decreased, which may represent impairment of cognitive functions (motor attention, language, executive function, etc.).

DEC variation increased significantly between the left IFG and the right MCG in the T2DM group, which is further evidence of decreased stability in the brain of T2DM. The frontal lobe is an important part of brain development and is responsible for higher-order cognitive control. Several studies (39–45) have shown that IFG, as the core region of the FPN, was associated with motor control, language processing, attention, and execution, which partially overlaps with the MCG’s functions. A study reported on decreased activation in the left IFG under low working memory load conditions in T2DM patients (46). In addition, patients with AD and MCI have reduced activation of IFG in several memory tasks compared with HCs (47). Eliasova found that regulation of IFG excitability by rTMS may result in improved attentional task performance in patients with early AD (48). It is therefore reasonable to speculate that both the MCG and IFG may be involved in the control of certain cognitive brain functions, such as language processing, attention, and working memory.

At the same time, to verify this point, this study included a variety of cognitive function-related scales to explore the changes of cognitive function in T2DM patients. The results of our study showed that the overall cognitive function of T2DM patients decreased to varying degrees, which is consistent with previous research (7). Compared with the HCs, the scores of MoCA, AVLT immediate recall, CDT, and GPT (R and L) scales decreased significantly. This further confirms that T2DM does lead to accelerated cognitive decline. According to previous studies, altered brain regions in T2DM have been linked to visual motor attention, language processing, and memory function (49–51). Accordingly, we also found evidence in the neuroimaging measurements. MoCA is a comprehensive test scale (52); its memory test designs more words and tasks, which are more demanding and more sensitive to detect MCI (53). It assesses executive function, language, and visual-spatial processing. Verbal memory decline is the main sign of brain aging and an important character of AD. AVLT which includes immediate recall, 5-min recall, 20-min recall, and recognition mainly tests verbal memory with high sensitivity (54). CDT detects executive function as well as visuospatial function (55). GPT asks subjects to place each specially shaped peg in a slot that fits it, which measures the performance speed of fine motor tasks (56). The decrease in these scores indicated an overall cognitive decline in T2DM patients.

**Figure 5:**
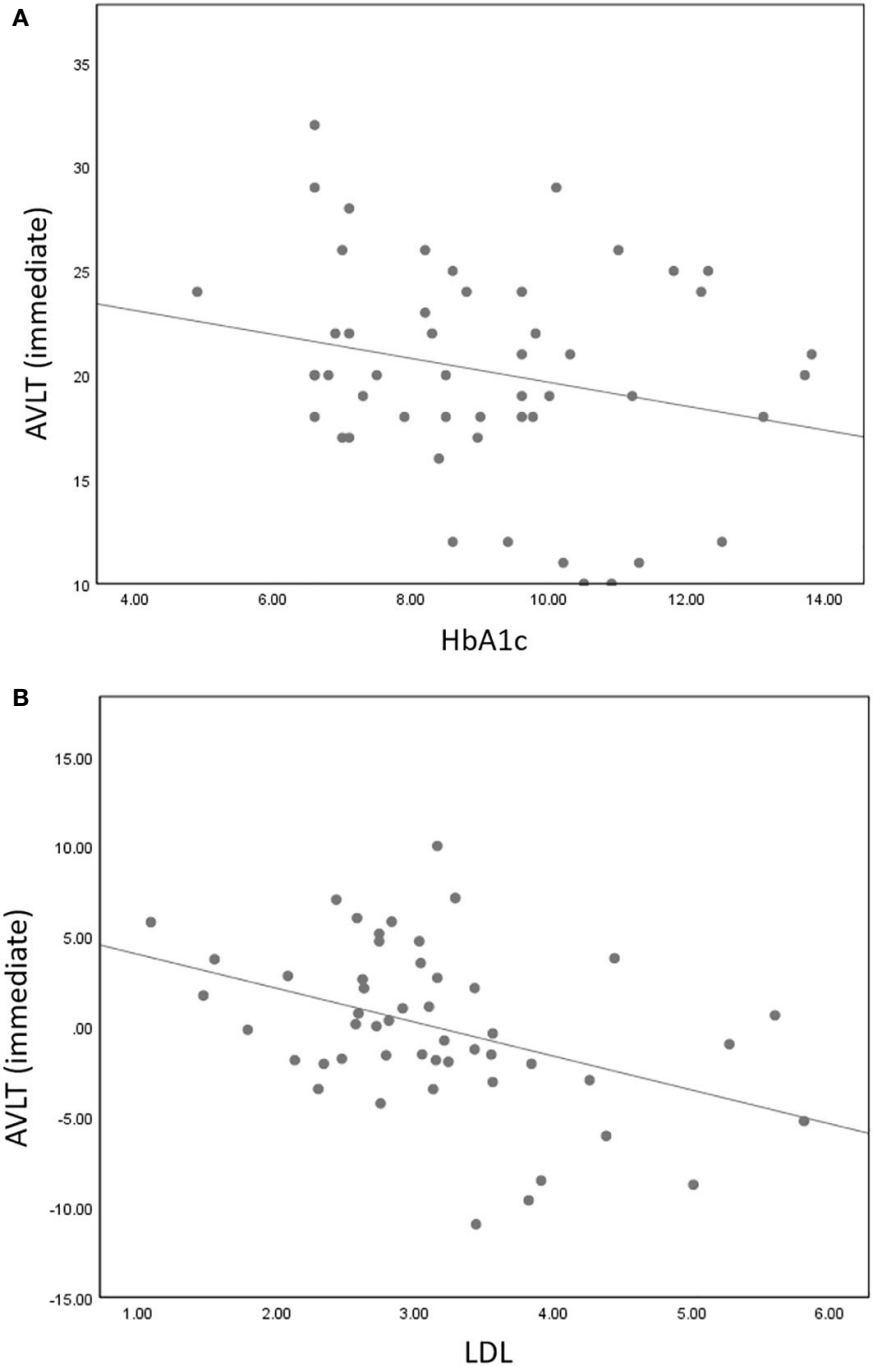
AVLT immediate memory scores were negatively correlated with HbA1c (r = -0.309, P = 0.035) **(A)** and LDL (r = -0.399, P = 0.004) **(B)**.

We also found a close association between clinical indicators and cognitive tests in the correlation analysis (**Figure 5**). As the LDL and HbA1c levels increased, AVLT scores decreased. A higher level of LDL was associated with worse memory function [spice] (57). Research shows that increased LDL level is an independent risk factor for MCI (58). Many studies have found a link between plasma lipids and AD (59–61). In the case of hyperlipidemia, an elevated plasma cholesterol level results in the formation of free radicals that can damage the blood–brain barrier, leading to an elevated cholesterol level in the brain (61). Cholesterol is crucial in the formation of amyloid in the brain, leading to the formation of more beta-amyloid plaques. Eventually, it leads to neurodegeneration (61). Similarly, several studies have shown that HbA1c is associated with reduced cognitive function in diabetics and suggests neurological damage (62–64). HbA1c represents the percentage of glycosylated hemoglobin and is considered the gold standard for controlling the efficacy of treatments for diabetes (64). There is strong evidence that HbA1c is negatively associated with cognitive function and structural integrity of the brain in diabetic patients without dementia (65, 66). This study suggested that in T2DM, decreased LDL and HbA1c levels may represent reduced cognitive function, especially memory function.

### 5.2 Methodological validity

Most of the previous studies used the AAL atlas of 116 regions to perform the atlas-to-atlas method. Since some clusters span multiple functional regions but are not visible using the AAL atlas, the atlas-to-atlas method is not detailed enough and may miss some details. The calculation of functional stability can be conducted by two ways containing “voxel to voxel” and “voxel to atlas.” The names of the two methods can manifest the accuracy level of measurement and the derivation process. In either method, the former “voxel” suggests that resolution of measurement and stability is measured voxel by voxel. The latter “voxel” suggests resolution of features, and it is at the voxel level. That is, for given voxel j, its dFC is computed with all other voxel across sliding windows and then stability is calculated. In the meantime, “atlas” suggests that the dFC is computed between voxel j and atlas. The “voxel to voxel” method is computationally accurate, but it is a huge amount of work and is time-consuming. We want to not only simplify the heavy workload but also ensure accurate measurement. The computation and results of the “voxel-to-atlas” method are very familiar to “voxel-to-voxel” so that it is the better way to perform the computation at a very fast rate. Therefore, the “voxel to atlas” method was used to calculate the stability. In Yan’s study, it is shown that the “voxel to atlas” method has high speed and precision (20). After comparing the two methods, the results showed that “voxel to atlas,” which is reasonable and feasible, could capture the main changes in the signal and the key information would not be lost. In this study, the “voxel to atlas” method was conducted to represent functional stability and the atlas with 200 regions was chosen that may make up for the shortcomings of past research. In addition, different window lengths and sliding steps were conducted to eliminate confounding effect.

## 6 Conclusion

In our study, changed dynamic functional architecture and directional connectivity in T2DM were explored and measured combining stability and dEC, which is an innovative attempt. Compared with HCs, T2DM subjects showed decreased stability in the right SMG and right MCG and dEC variation has increased significantly between left IFG and right MCG. The direction of causal flow is from the right MCG to the left IFG. These results provide additional evidence for cognitive decline in T2DM.

## 7 Limitation and future expectations

There was no correlation between neuroimaging biomarkers and cognitive function scores. We speculate that the sample size is relatively small, and the correlation may not be linear. In the future, we will continue to increase the sample size and reveal the relationship between neuroimaging indicators (dynamic stability and dEC) and cognitive function scores by multiple statistical methods. In addition, because the onset of T2DM is hidden and the specific time of onset is not precise enough, the course of the disease was not included in the study. In future studies, a very accurate disease course will be concluded.

## Data availability statement

The raw data supporting the conclusions of this article will be made available by the authors, without undue reservation.

## Ethics statement

This protocol was approved by the Ethics Committee of Guangzhou University of Chinese Medicine (Approval number: K2020115). Each subject provided written informed consent according to the Declaration of Helsinki. The patients/participants provided their written informed consent to participate in this study.

## Author contributions

YFL designed the whole experiment and completed the manuscript. ML was involved in data processing. KZ and YW participated in the analysis design. XT and CQ recruited the study subjects. YR, ZS, LG, and ZC contributed to the statistical analysis. YL and SQ revised the manuscript. SQ is the guarantor of this work and, as such, had full access to all the data in the study and takes responsibility for the integrity of the data and the accuracy of the data analysis. All authors contributed to the final version of the manuscript and approved the final manuscript.
