# Supplementary material for: Altered dynamic functional architecture in type 2 diabetes mellitus
Source: Front Endocrinol (Lausanne). 2023 Jan 24;13:1117735. doi: 10.3389/fendo.2022.1117735 (PMC9903314; doi:10.3389/fendo.2022.1117735)
Supplement: Supplementary file 1 [file DataSheet_1.pdf]

Supplementary Material

Stability of brain dynamic functional architecture in type 2 diabetes mellitus

Yifan Li [1,2†], Mingrui Li[1,2,3†], Kui Zhao [1,2], Yan Wang [1,2], Xin Tan [2], Chunhong Qin [2], Yawen Rao [1,2], Zhizhong Sun [1,2], Limin Ge [1,2], Zidong Cao [1,2], Yi Liang [1,2*], Shijun Qiu[1,2*]

*** Correspondence:**

Prof. Shijun Qiu, MD, Ph.D; qiu-sj@163.com

Prof. Yi Liang, MD, Ph.D; lysogood@126.com

1 Supplementary Figures and Tables

Supplementary Figure 1

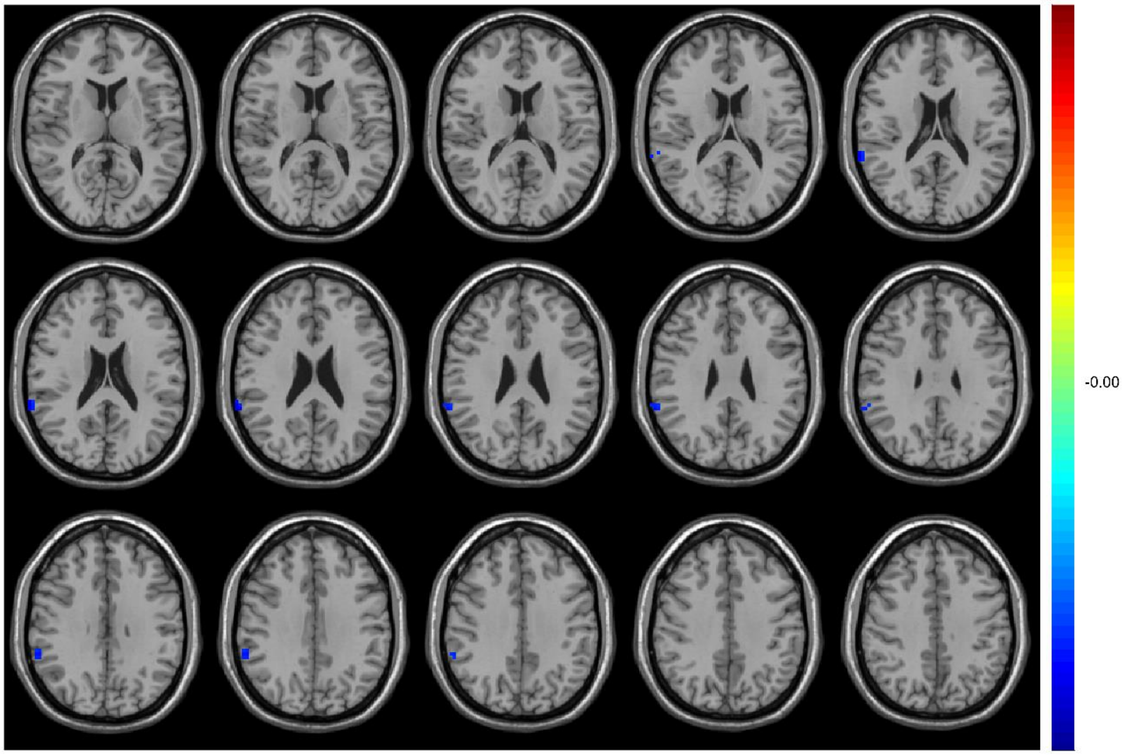

Compared with HCs, T2DM subjects exhibited decreased stability in the right supra-marginal gyrus and right medial cingulate gyrus with window width of 60s and step size of 2s.

Supplementary Figure 2

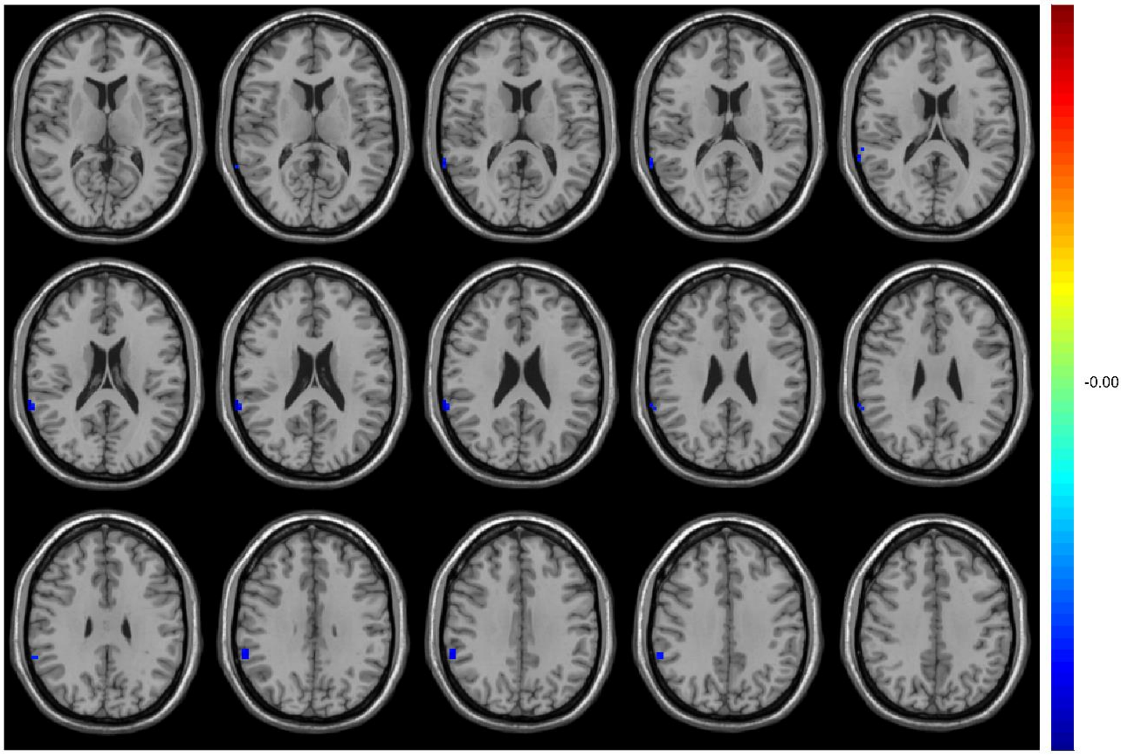

Compared with HCs, T2DM subjects exhibited decreased stability in the right supra-marginal gyrus and right medial cingulate gyrus with window width of 96s and step size of 8s.

Supplementary Table 1

Brain regions with altered stability in T2DM subjects using different window width and step size

Window width/Step size(s)	Cluster	Brain regions	MNI coordinates			Voxels	t-value
			x	y	z		
60/2	1	Right Supra-Marginal gyrus	69	-42	21	30	-4.5062
	2	Right Median Cingulum gyrus	3	-12	48	25	-4.71

96/8	1	Right Supra-Marginal gyrus	69	-42	21	31	-4.7134
	2	Right Median Cingulum gyrus	3	-12	48	25	-4.8477

MNI, Montreal Neurological Institute; X, Y, and Z, coordinates of primary peak locations in MNI space.
